# The strategies of perioperative management in orthopedic department during the pandemic of COVID-19

**DOI:** 10.1186/s13018-020-01978-y

**Published:** 2020-10-15

**Authors:** Hui Zeng, Guoqing Li, Jian Weng, Ao Xiong, Chang Xu, Yifei Yang, Deli Wang

**Affiliations:** 1 Department of Bone & Joint Surgery, Peking University Shenzhen Hospital, Shenzhen, 518036 People’s Republic of China; 2 National & Local Joint Engineering Research Center of Orthopaedic Biomaterials, Department of Bone & Joint Surgery , Peking University Shenzhen Hospital, Shenzhen, 518036 People’s Republic of China; 3 Department of Medical Administration, Peking University Shenzhen Hospital, Shenzhen, 518036 People’s Republic of China

**Keywords:** Coronavirus disease 2019 (COVID-19), Orthopedic surgery, Perioperative management, Flow chart

## Abstract

**Background:**

Coronavirus disease 2019 (COVID-19) has broken out and spread rapidly nationwide at the beginning of 2020, which has brought huge impacts to people and work. The current situation of prevention and control is severe and urges guidance for clinicians, especially for medical systems. In the hope of providing a reference and recommendation for the prevention and control of the COVID-19, we carried out research to improve the quality of patient care and prevention during this epidemic.

**Methods:**

All of the staff were trained rapidly to master personal protection in our department. We reviewed the patients’ discharged records who underwent surgery in our department during January 1 to March 1, 2019, and January 1 to March 1, 2020. The management of the surgery patients and flow charts were described and analyzed. Post-operation outcomes of the patients include duration, complications, surgical site infection (SSI), system infection, re-operation, and mortality. Both chi-squared test and Student’s *t* test were performed to determine the relationship between the two periods in terms of post-operation outcomes.

**Results:**

Descriptive statistics analysis revealed that demographic of the patients between the two periods is similar. We had benefited from the strict flowcharts, smart robot, and protection equipment during the perioperative managements for orthopedic patients. With the help of the strict flow charts and smart equipment, post-operation outcomes of the patients revealed that the rates of the complications and re-operation had been reduced significantly (*p* < 0.05), while duration of operation, SSI, and system infection had no significant difference between two periods (*p* > 0.05). No patient and staff caught COVID-19 infection or mortality during the epidemic.

**Conclusions:**

Our study indicated that medical quality and efficiency were affected little with the help of strategies described above during the epidemic, which could be a reference tool for medical staff in routine clinical practice for admission of patients around the world. What is more, the provided strategies, which may evolve over time, could be used as empirical guidance and reference for orthopedic peers to get through the pandemic and ensure the normal operation of the hospital.

## Introduction

Since December 2019, an unexplained type of coronavirus has appeared rapidly in Wuhan followed by bronchitis and pneumonia [1, 2]. It has been proven that the pathogen was coronavirus disease 2019 (COVID-19), which seriously threatens surgical staff and patients, and challenged the medical community to an unprecedented degree [3]. COVID-19 can induce not only mild to severe respiratory diseases, but also inflammation, high fever, cough, acute respiratory tract infection, and dysfunction of internal organs which may result in death [4]. The World Health Organization (WHO) declared it as a pandemic on 11 March 2020 because of its rapid worldwide spread [5]. The public health emergency caused by the pandemic has resulted in a significant reallocation of health resources with a consequent reorganization of the clinical activities in orthopedic department.

As secondary transmissions have occurred and the speed of transmission is accelerating, there are rising concerns about community infections and the overwhelming majority of cities have launched higher level response [6, 7]. The current situation of prevention and control is grim. Some of orthopedics have agreed on possible strategies for the reorganization of orthopedic routine practice and on a set of recommendations that should facilitate the process of rescheduling both inpatient and outpatient activities during the pandemic.

The epidemic of COVID-19 poses new challenges to diagnosis and treatment of the patients with orthopedic diseases [8]. We medical workers are bearing important responsibilities and pressure during the epidemic. Orthopedic surgeons performed the superiority of accurate diagnosis and treatment for patients, summarized how to carry out the clinical practice of orthopedic surgery under the situation of the prevention and control of the COVID-19, and minimized the risk of infection exposure. Reasonable treatment strategies were changed and adopted timely to minimize the adverse effects on the treatment of orthopedic patients during the epidemic.

The staff in our department make every effort in making correct diagnosis and treatment of specialized diseases by optimizing process, providing proper medical advice, and mastering indications of selective, confine, and emergency operation reasonably. Based on the full understanding of the characteristics of orthopedic diseases and COVID-19, in order to summarize and discuss available evidence for orthopedic practices, we provide the highest quality medical services in the form of flow charts as for the regular clinical practice.

## Methods

### Study sample and patients

We built a dataset containing 96 patients who underwent operation between January 1 to March 1, 2019, and January 1 to March 1, of 2020. Data were sourced retrospectively from the medical records. Each discharged abstract involved a multitude of variables. Demographics of the patients of the two periods were described and analyzed, and analysis revealed that they are similar (Table 1). Ethical review approval was obtained from the Human Subject Committee at Ethics Committee of Peking University Shenzhen Hospital (Ethics Committee of Peking University Shenzhen Hospital (research) [2020] 013th).

**Table 1:** Analysis of patient demographic characteristics of the two periods (*N* = 96)

Categories	Unit	2019 (*N* = 50)		2020 (*N* = 46)	
		Count	Percentage	Count	Percentage
Patients	Percentage	50	52.08%	46	47.92%
Age	Year	–	61.48 ± 17.77	–	52.11 ± 21.33
Gender	Male	22	22.92%	23	23.96%
	Female	28	29.17%	23	23.96%
Diagnosis	Fracture	45	46.88%	41	42.71%
	Infection	1	1.04%	2	2.08%
	Tumor	0	0	1	1.04%
	Deformity	1	1.04%	0	0
	Degeneration	0	0	1	1.04%
	Others	3	3%	1	1.04%
Anesthetization	General	21	21.88%	11	11.46%
	Non-general	29	30.21%	35	36.45%
ASA class	1	1	1.04%	7	7.80%
	2	0	0	1	1.04%
	3	38	40.58%	26	27.08%
	4	11	11.45%	12	12.50%

*ASA* American Society of Anesthesiologists

We reviewed many variables noted in the literature, and a set of recommendations and flow charts were created based on a review of the communications for surgeons with knowledge of safety procedures.

We recommend inpatients during hospital stay should be provided with as many instructions as possible to stay in wards. Patients should be evaluated in detail under stable condition to minimize the risk of readmission. It is advisable for us to reduce or reschedule post-discharge controls and implement an adequate system of communication for telemonitoring patients in order to reduce hospital visits.

In the hope of providing a reference and recommendation for the prevention and control for surgeons, we share our experience during the epidemic in the form of flow charts.

### Statistics analysis

Both chi-squared test and Student’s *t* test were performed to determine the relationship between the two periods. For all analyses, *p* < 0.05 was considered statistically significant. All statistical analyses were performed on IBM SPSS Statistics (version 23.0).

### Practical flow charts

By summarizing the research progress and guidelines in recent years in the fields of orthopedic diseases, treatment strategies and perioperative management were developed to provide more choices for patients to obtain the best treatment under the severe epidemic. COVID-19 brought huge impacts to people and work nationwide; the routine diagnosis and treatment of fractured patients were affected with varied degrees as well.

Instead of traditional diagnosis and treatment, a new system should be developed. Simplification of the diagnostic and staging pathway has to be prioritized in order to reduce hospital visits and consequently the risk of contagion. Orthopedic is not a front-line subject, but fracture is a common injury, and most of the patients are in trouble when diagnosed during the epidemic [9, 10]. The regular diagnosis and treatment of patients were greatly affected, and elective surgical activity of the hospital was rapidly reduced.

**Fig. 1:**
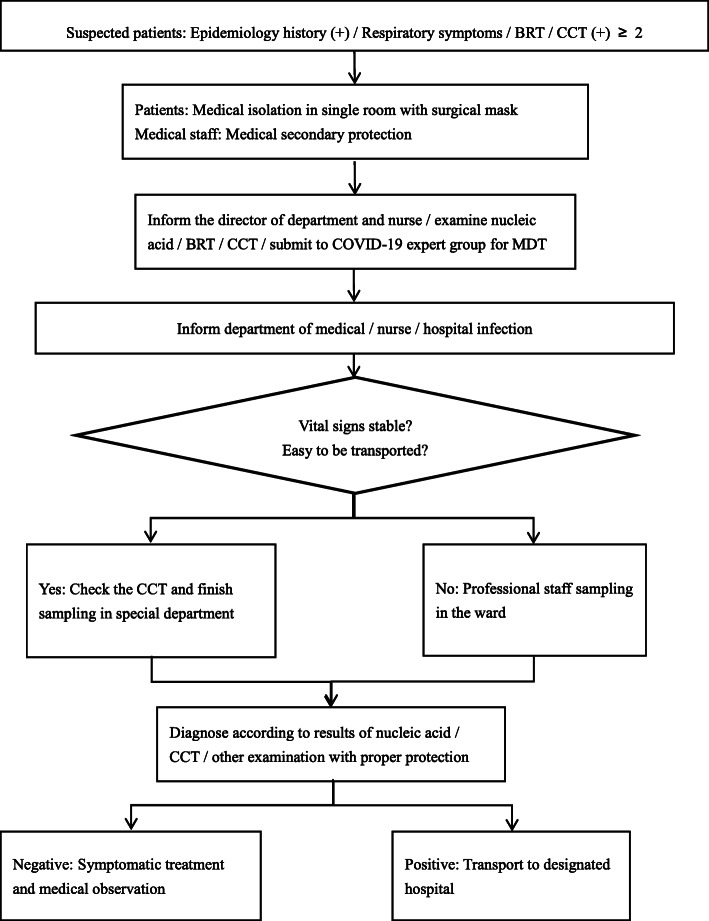
Flow chart of suspected patient identification

**Fig. 2:**
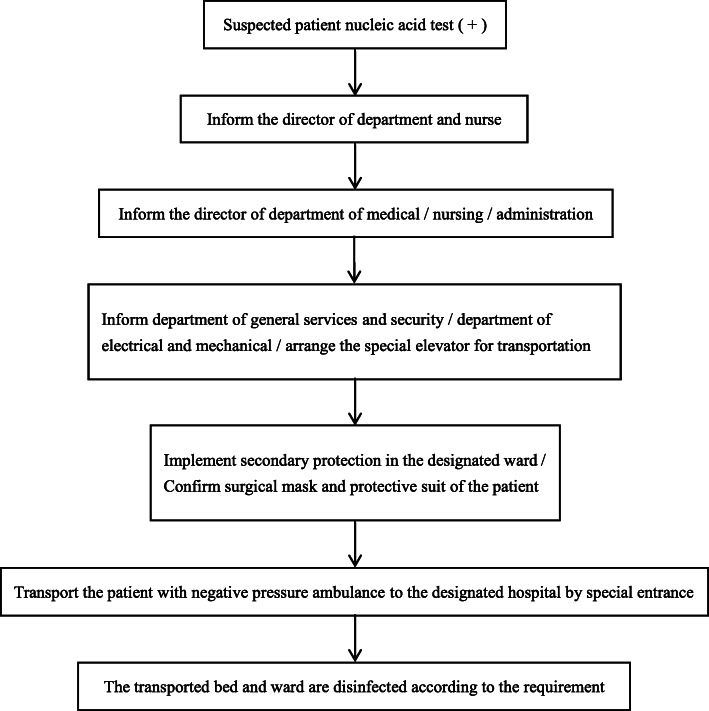
Flow chart of patients who were identified positive for nucleic acid test

Meanwhile, we hope that it could provide more treatment model schemes for colleagues and share the flow charts of management for patients during the breakout of pandemic, including prevention and control measures for staff, operating rooms, and surgical instruments, which may be beneficial for medical staff (Fig. 1). Meanwhile, we prepared workflow for patients who were diagnosed with COVID-19 (Fig. 2).

**Fig. 3:**
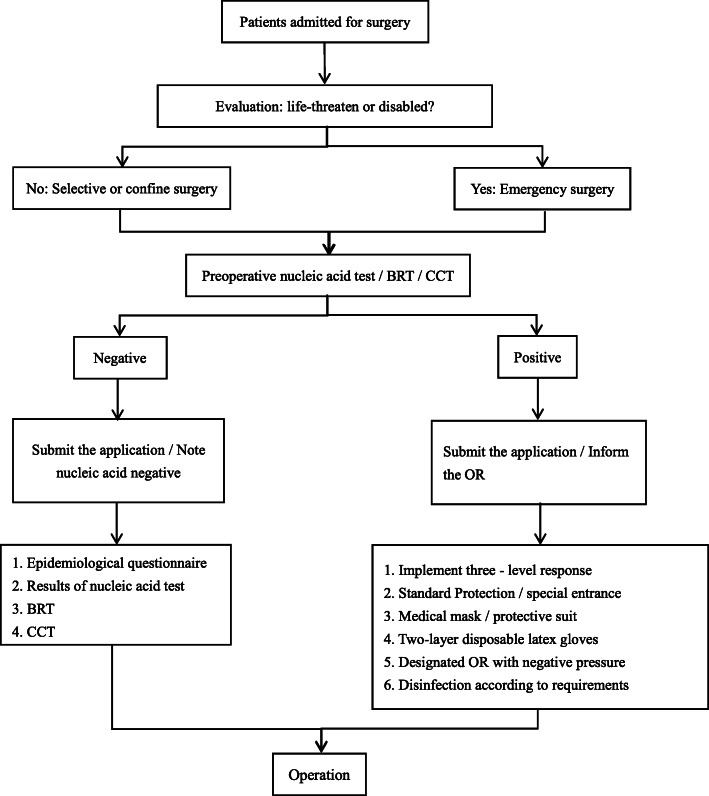
Flow chart of surgery management for inpatients

Moreover, most of parts were involved during the special epidemic. As parts of epidemic response measures, the selection of surgical procedures and perioperative management of orthopedic diseases require all staff to work together to figure out a reasonable system of surgical treatment and emergent response. We concluded experience as following and shared the experiences of the management of patients who are scheduled or emergent to be admitted (Fig. 3).

**Fig. 4:**
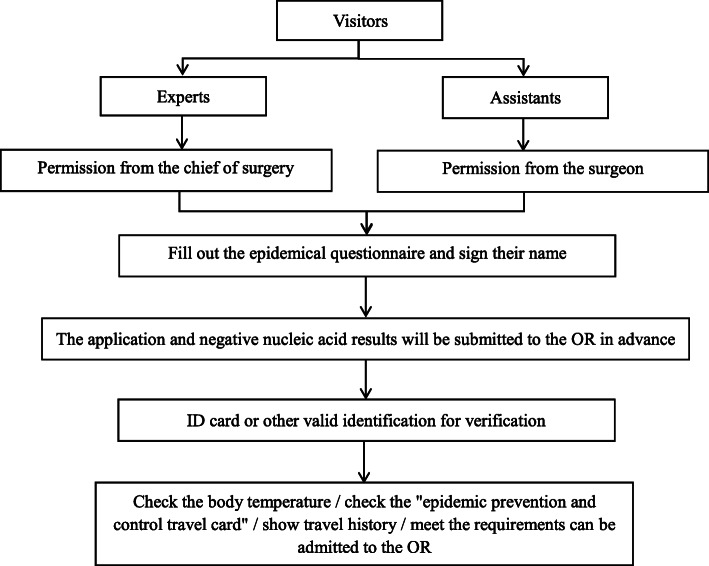
Flow chart of management for visitors

Some of the surgeons and assistants who come from outside of Shenzhen but ought to enter the OR should obey the special flow chart as well (Fig. 4).

**Fig. 5:**
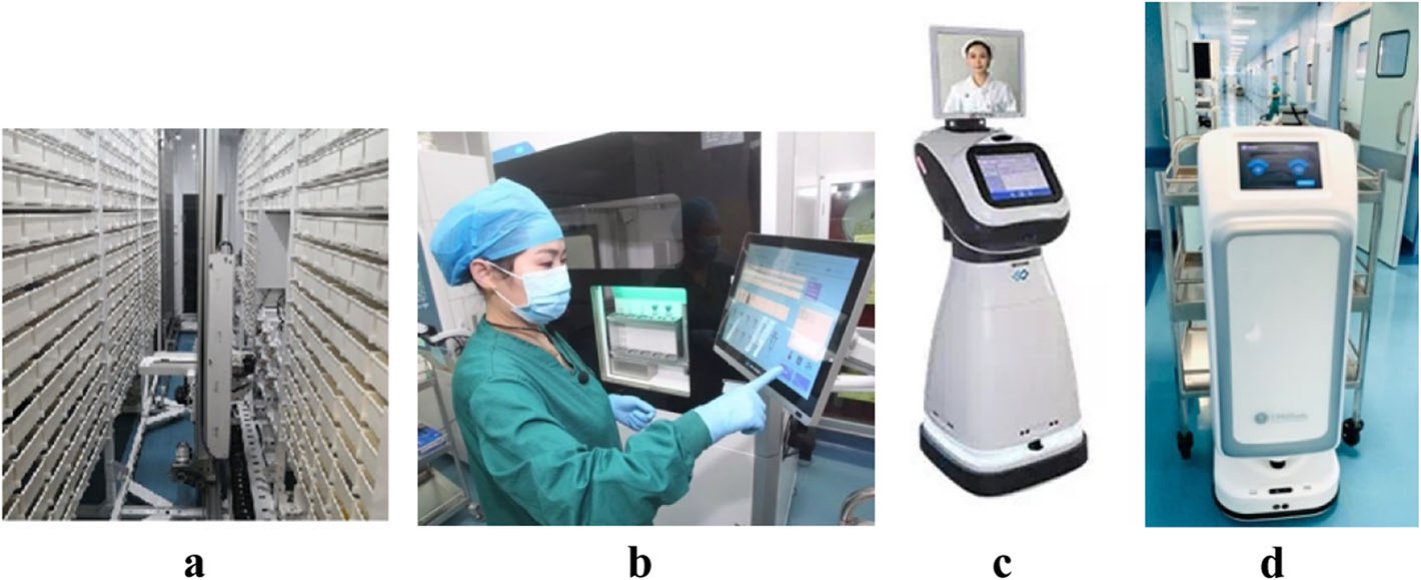
Smart assistant robots (**a**–**d**)

Behavioral assistance robot is withdrawing medicine in the pharmacy (Fig. 5a), which can meet the requirements of a wide variety of clinical drugs and reduce the workload or contact and improve the accuracy. Manually monitoring can ensure the entire process is rigorous, accurate, standardized, and safe, which can completely eliminate the inevitable problems of poisoning and pollution in manual operation (Fig. 5b). Nurse assistant robot looks like a cartoon nurse (Fig. 5c), which can assist nurses to complete routine nursing tasks, such as procedure handling, environmental guidance, data query, ward inspections, item delivery, and data printing. Robots transport (Fig. 5d) sterile materials in the OR without touching. Smart assistant robots can help staff in many areas, which are always on call and tireless. With the help of these smart robots, the staff have saved much energy and reduce the contact with patients.

**Fig. 6:**
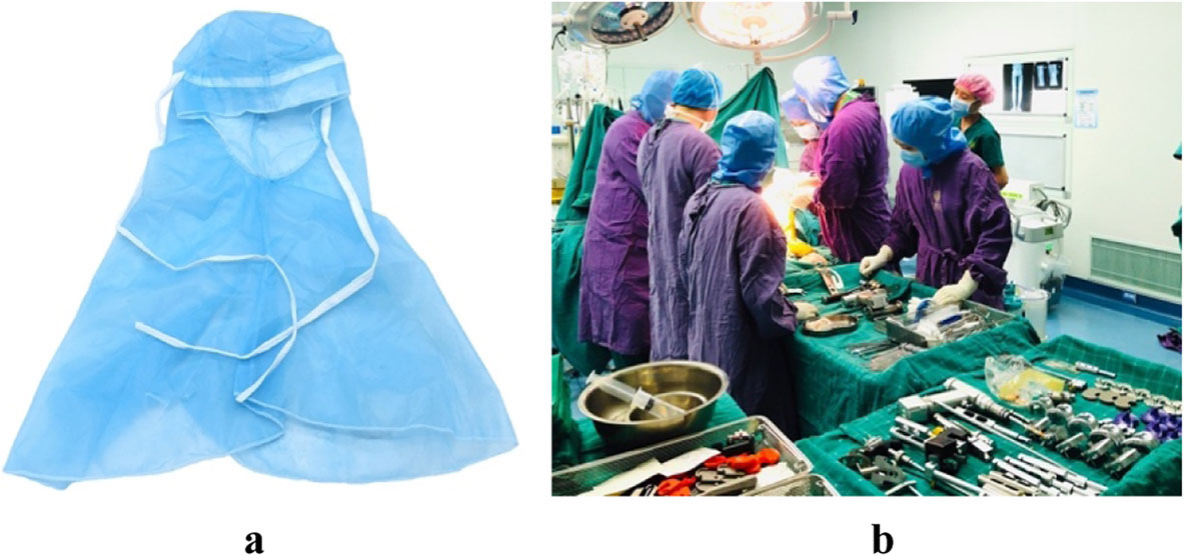
Special hat (**a**) and TKA intraoperative photo (**b**)

All of the staff were trained rapidly to face the epidemic such as attaching great importance to the personnel protection and implementing the relevant rules and regulations for prevention strictly. One of the personal protection equipment is special surgery hat (Fig. 6a), which can help surgeons reduce the contact and exposure during the operation. Intraoperative photo of selective surgery of total knee arthroplasty (TKA) with special hat is shown in Fig. 6b.

## Results

Analysis for post-operation outcomes of the two periods was displayed (Table 2).

**Table 2:** Analysis of patient outcomes between the two periods (*N* = 96)

Patient outcomes	2019 (*N* = 50)		2020 (*N* = 46)		*t*/*χ*^2^	*P* value
	Count	Percentage	Count	Percentage		
Duration (min)	205.9	–	201.3	–	0.591	0.261
Complications	6	6.25%	0	0%	5.888	0.015*
Re-operation	4	3.13%	0	0%	3.84	0.049*
SSI	1	1.04%	1	1.04%	0.004	0.952
System infection	1	1.04%	0	0%	0.93	0.335
COVID-19 infection	0	0%	0	0%	–	–
Mortality	0	0%	0	0%	–	–

*SSI* surgical site infection

*p < 0.05, statistically significant

We can tell from the results of the two periods that no staff or patient caught infection of COVID-19 during the epidemic, or no mortality of the two periods. With the help of the strict work flow and smart equipment, the rates of adverse outcomes including complications and re-operation had been reduced significantly compared to the former year (*p* < 0.05), and patients who underwent surgery may have benefited from the work flow during the pandemic. However, duration, SSI, and system infection had no significant difference between the two periods (*p* > 0.05).

## Discussion

COVID-19 could result in killing quantities of people, and the current situation of prevention and control is severe. Critical patients are often occures in the old patients who caught multiple comorbidities or lack of health care, which could progress to acute respiratory distress syndrome (ARDS), multi-organ dysfunction (MODS), or even death [11, 12]. We searched the literature and assessed the certainty in the evidence using the recommendations based on the experience of health care systems in Asia and Europe [13], and recommendations were in the form of best clinical practice. Furthermore, many relevant steps were adopted and clinical activities were carried out to explore new strategies and effective therapies during the epidemic.

It is particularly important to underline the clinical features of COVID-19, especially in the early stage of the illness. Up to now, multiple guidelines have been issued by various organizations to recommend their practice. In relationship with orthopedic diseases during the epidemic, related prevention and control, clinical recommendations, diagnosis and treatment, clinical management, health care personnel protection, and disinfection were applied soon in our hospital.

National recommendations and local infection control guidelines are tailored to the availability of medical resources, which were imminently adopted to fight against the pandemic [14, 15]. de Girolamo et al. believe that nonsteroidal anti-inflammatory drugs (NSAIDs) may induce increased sensitivity to more severe clinical features in coronavirus infection [16]. The Department of Oral and Maxillofacial Surgery of Peking University School and Hospital of Stomatology shared their experience. They make proper diagnosis and treatment for patients according to the urgency and severity of the disease and interventions by following the flow chart strictly as well [17]. Luo and Zhong introduced details of Renji experience as for parts of general surgery [18]. Italian urologists recommended on pathways of perioperative care for urological patients undergoing urgent procedures, which may be inspired for urological societies [19]. Anesthesiologists are required to adopt tailored anesthetic practices to individual patients which will ensure the best outcomes [20]. Former researchers provide the views of related diseases, and they believed that strategies were suitable for physicians, which is good for both patients and the perioperative management team.

The health care personnel of department of orthopedics are vulnerable to the infection due to their extensive and close exposure to patients. In consideration of the rapid spread of epidemic, health care staff are at added risk of exposure and infection during the practice of treatment [13]. For the purpose of limiting the quantity of the people and reducing the chance of contacting, physical therapy should be provided only to immediate post-operative patients and some of the other proper patients should be replaced by home exercise programs [21]. The treatment strategy should be changed timely, and appropriate methods should be adopted to minimize the adverse effect of the epidemic in orthopedic diseases’ treatment.

It is a difficult task how to maximize the protection for health of medical staff, and the safety of wards and hospitals. In our study, none of staff was infected during the clinical practice. Preserving a highly skilled health care workforce is a top priority for any community and health care system. To avoid the aggravation of COVID-19 or collapse of the health system, emergency department was emphasized in our hospital and several recommendations were issued to help support health care workers against the pandemic.

For the purpose of control COVID-19, we conclude the experience of our department during the pandemic. First of all, strict and detailed work flow chart could be a clear guidance for medical staff in various situations. Secondly, variable departments should cooperate together for special period to optimize the medical service and improve quality and efficiency. Thirdly, we staff should pay more attention to the construction of hospital artificial intelligence and high technology information such as make full use of the smart assistant tools, which may be a key solution to the prevention and control of the epidemic.

There are several limitations in our study. First, we conducted this retrospective cohort study by using a database in our department within 2 years, particularly with new generation constructs. Prospective studies are needed to validate these calculators and refine over time. Moreover, the database was small and did not include information on long-term follow-up outcomes. Last but not least, the provided flow charts, which may evolve over time, could be used as guidance for health care workers who are involved in the care of patients. When available, we will provide new evidence in further releases of these guidelines and we believe that future studies are necessary to define more flow charts briefly and clearly.

## Conclusion

Our study indicated that medical quality and efficiency were affected little with the help of strategies described above during the epidemic, which could be a reference tool for medical staff in routine clinical practice for admission of patients around the world. What is more, the provided strategies, which may evolve over time, could be used as empirical guidance and reference for orthopedic peers to get through the pandemic and ensure the normal operation of the hospital.

## Data Availability

Please contact the author for data requests.

## Abbreviations

BRT: Blood routine examination
CCT: Chest computed tomography
MDT: Multidisciplinary diagnosis and treatment model
COVID-19: Coronavirus disease 2019
OR: Operating room
ID card: Identification card
SSI: Surgical site infection
TKA: Total knee arthroplasty
ASA: American Society of Anesthesiologists
NSAIDs: nonsteroidal anti-inflammatory drugs
ARDS: Acute respiratory distress syndrome
MODS: Multi-organ dysfunction
